# Automatic Prediction of Ischemia-Reperfusion Injury of Small Intestine Using Convolutional Neural Networks: A Pilot Study

**DOI:** 10.3390/s21196691

**Published:** 2021-10-08

**Authors:** Jie Hou, Runar Strand-Amundsen, Christian Tronstad, Jan Olav Høgetveit, Ørjan Grøttem Martinsen, Tor Inge Tønnessen

**Affiliations:** 1 Department of Physics, University of Oslo, Sem Sælands vei 24, 0371 Oslo, Norway; jieho@fys.uio.no (J.H.); jhogetve@ous-hf.no (J.O.H.); 2 Department of Clinical and Biomedical Engineering, Oslo University Hospital, 0372 Oslo, Norway; runar@sensocure.no (R.S.-A.); chrton@ous-hf.no (C.T.); 3 Department of Emergencies and Critical Care, Oslo University Hospital, 0424 Oslo, Norway; t.i.tonnessen@medisin.uio.no; 4 Institute of Clinical Medicine, University of Oslo, 0424 Oslo, Norway

**Keywords:** convolutional neural networks (CNN), small intestinal viability, ischemia-reperfusion injury, explainable AI, transfer learning, probabilistic modeling, decision-level fusion

## Abstract

Acute intestinal ischemia is a life-threatening condition. The current gold standard, with evaluation based on visual and tactile sensation, has low specificity. In this study, we explore the feasibility of using machine learning models on images of the intestine, to assess small intestinal viability. A digital microscope was used to acquire images of the jejunum in 10 pigs. Ischemic segments were created by local clamping (approximately 30 cm in width) of small arteries and veins in the mesentery and reperfusion was initiated by releasing the clamps. A series of images were acquired once an hour on the surface of each of the segments. The convolutional neural network (CNN) has previously been used to classify medical images, while knowledge is lacking whether CNNs have potential to classify ischemia-reperfusion injury on the small intestine. We compared how different deep learning models perform for this task. Moreover, the Shapley additive explanations (SHAP) method within explainable artificial intelligence (AI) was used to identify features that the model utilizes as important in classification of different ischemic injury degrees. To be able to assess to what extent we can trust our deep learning model decisions is critical in a clinical setting. A probabilistic model Bayesian CNN was implemented to estimate the model uncertainty which provides a confidence measure of our model decisions.

## 1. Introduction

Acute intestinal ischemia is a serious condition with a high mortality rate, where rapid diagnosis and treatment are of crucial importance [1,2]. With a positive diagnosis, the goal of the acute surgery is to assess the small intestine, restore blood flow, and resect segments that appear to be non-viable while leaving the intestinal segments that will ultimately survive [1]. Diagnosing intestinal ischemia intra-operatively is difficult, especially in circumstances where the bowel appears to be in the borderline between ischemic and non-ischemic [1].

The standard clinical method for evaluation of intestinal viability is still visual inspection and palpation. The estimation of tissue state can be based on color change, presence of visible peristalsis and bleeding from cut edges [1,2,3,4,5]. This method is non-specific and often unreliable. Viable tissue might be removed or more importantly, irreversibly injured tissue might be left in the patient, which may lead to complications and slow down the patient’s recovery. For standard clinical estimation of bowel viability, reports have been made of accuracy in the range of 78% to 89%, but this typically includes resection of viable bowel and second-look procedures [5,6]. Patients may risk short gut syndrome if resection is performed too aggressively. Techniques, such as anti-mesenteric Doppler interrogation and intravenous fluorescein dye, have been used experimentally. However, none of these techniques have proven to be reliable in predicting future viability of the small intestine [2]. Due to the potential inaccuracy of the early evaluation of intestinal viability following ischemia-reperfusion injury, a second-look operation, 24–48 h after the first surgery, is often required [1,2]. Prolonged or repeated surgery increases the amount of surgical stress and the risk for infection. A quick and accurate technique for intraoperative evaluation of intestinal viability could reduce the need for second-look operations and the time needed for evaluation.

CNN is a well-known deep learning model for image analysis and classification. In this study, we investigated four different CNN architectures; traditional CNN, CNN with decision-level fusion, Bayesian CNN, and transfer learning using ResNet50 [7]. Decision-level fusion CNN was chosen as fusing multiple image datasets enhances the classification confidence and reduces ambiguity and uncertainty. Moreover, since the traditional CNN architecture does not offer information about how trust-worthy the model is, i.e., uncertainty in its predictions or decisions, we further chose to apply a probabilistic model, Bayesian CNN architecture, which has the ability to quantify the model’s reliability. In a clinical setting, not only does the diagnostic process by machine learning models have to be extremely accurate, but knowing to what extend we can trust our model decisions is also crucial. The chosen Bayesian CNN architecture is robust to over-fitting and at the same time offers a probabilistic interpretation of the model by inferring distributions over the model’s weights [8]. We also explored the possibility of using transfer learning, where a developed model based on one task is reused as the starting point for a model with a similar dataset and task. We chose ResNet50, as this network has learned rich feature representations for a wide range of images, which can be of advantage for our task.

In addition to the machine learning models, two parameters from image analysis were also explored to quantify changes of properties in the images during ischemia and reperfusion. Namely, contrast for the red channel and entropy of the images. The results were further compared with the explainable AI Shapley additive explanations (SHAP) method, which is an approach to explain the output of any machine learning model [9]. This is demonstrated by highlighting pixels on the small intestine images to reveal which areas that contribute positively and negatively to the correct classification.

To the best of our knowledge, no previous studies have been conducted to investigate whether microscopic images taken from the surface of the intestinal tissue, combined with deep learning, can be used as a non-invasive method to evaluate the viability of the small intestine. The closest “state of the art” works, which use machine learning to assess diagnosis of gastrointestinal diseases, are multi-class image and video datasets for gastrointestinal endoscopy [10]. They demonstrated the potential benefits of AI-based computer-assisted diagnostic systems. In the same year, Yan et al. [11] used CNN and transfer learning in diagnosing gastric intestinal metaplasia with a limited number of images. In our study, we propose a fast and non-invasive method to assess small intestinal viability. We aim to investigate the use of images combined with deep learning algorithms to automatically evaluate the intestinal viability with millimeter precision during surgery.

## 2. Methods

### 2.1. Animals and Experimental Design

This study was conducted using ten Norwegian Landrace pigs, weight range 50 to 66 kg. Food was withheld 12 h prior to surgery. We used the same experimental protocol as Strand-Amundsen et al. [12]. For each pig, we created 4 segments with local ischemia on the jejunum, by clamping the arteries and veins of a segment of the jejunal mesentery. The result was a 30 cm central zone of warm ischemia and two surrounding edge zones of marginal tissue hypoxia. Images were acquired over an eight hours period. The 4 segments in each pig were used to create 4 different cases; case A: control 8 h; case B: 8 h ischemia; case C: 3 h ischemia followed by 5 h reperfusion; case D: 4 h ischemia followed by 4 h reperfusion. Images were taken once an hour. After the experiment, the animals were sacrificed by a lethal dose of potassium chloride (100 mmol).

### 2.2. Surgery, Anaesthesia, and Monitoring

Prior to surgery, the pigs were sedated by an intramuscular injection consisting of ketamine (40 mg/kg), atropine (0.05 mg/kg), and droperidole (0.65 mg/kg). During surgery, anesthesia was maintained with isoflurane (Abbott Scandinavia AB, Kista, Sweden) (1–1.5%) and a mixture of air and O${}_{2}$ to obtain an FiO${}_{2}$ of 30%. Morphine (Alpharma, Oslo, Norway) 0.4–0.7 mg/kg/h was administered as a continuous intravenous infusion. Surgery was performed under sterile conditions. The jejunum was made accessible through midline laparotomy in the abdominal cavity. A continuous infusion of Ringer acetate 10–30 mL/kg/h was administered through the jugular vein as fluid replacement [12].

### 2.3. Image Acquisition

**Figure 1:**
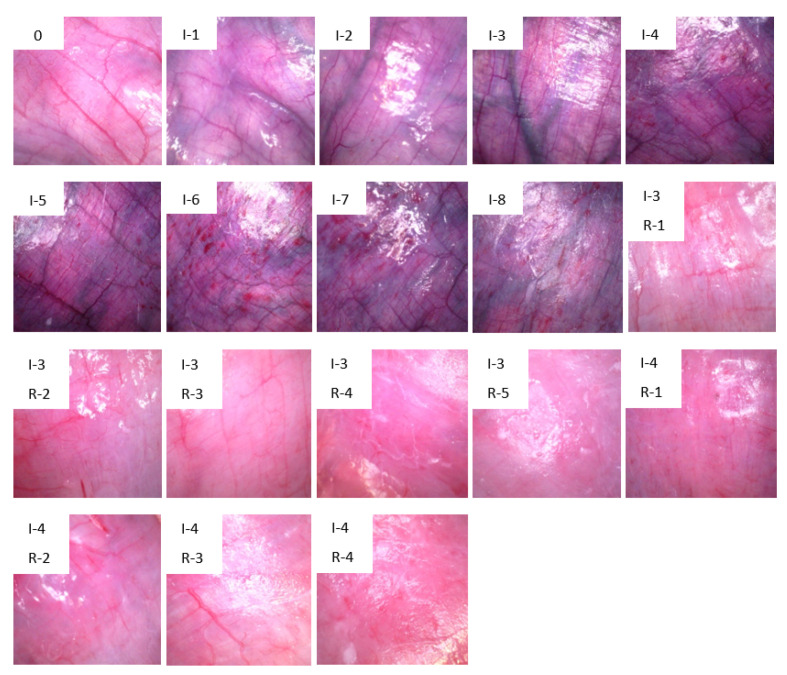
Images of the jejunum at selected intervals of ischemia and reperfusion. 0: healthy jejunum at the start of the experiment. “I” = Ischemia and “R” = Reperfusion, numbers are duration hours. Each of these 18 images represents distinct time duration’s that we use as classes in the CNNs.

A handheld Dino-lite USB digital microscope camera (model AM7013MZT from Dino-lite) with a controlled built-in light source was used to acquire images of the small intestine surface. To ensure that all images were taken under the same light conditions, a 3D printed black enclosure was used to limit external lights from influencing the area of interest. A series of images were taken at different locations of all 4 segments once an hour. Around 50 images were taken from each of the segments by moving the camera slightly after each image, resulting in 200 images per hour and 1600 images for each experiment. The total number of images acquired from 10 pigs was 17,330, divided into 18 classes based on one hour interval as shown in Figure 1.

### 2.4. Image Preprocessing

The acquired images are of size 2592 × 1944 pixels. As a part of the image preprocessing process, the edges of the images were first cropped automatically using the function “PIL.Image.crop()” from the Python Imaging Library [13] to eliminate background noise, to a new size of 1500 × 1400 pixels. To allow the 17,330 images to be of a manageable size relative to available computer memory, the images were further downsized to an image size of 224×224 pixels, while still retaining enough information, and to match the acceptable image size for the ResNet50 network.

After cropping the image edges and resizing them, the StandardScaler algorithm was implemented by:

(1) $$z=\frac{x-\mu }{\sigma }$$

where μ is the mean and σ is the standard deviation. Normalizing the training data to having mean 0 and variance 1 along the features can often improve convergence during gradient descent, since it will avoid many extra iterations that are required if one or more features have much larger values than the rest.

### 2.5. Entropy and Contrast Analysis

In order to investigate different image properties, we derived descriptive statistics for image entropy, contrast, dissimilarity, homogeneity, energy, and correlation. Among the properties investigated, the largest changes were found for entropy and contrast, and the development in these parameters during ischemia and reperfusion are presented statistically.

Shannon entropy [14] was calculated for each of the images, which is a direct measure of the number of bits needed to store the information in a variable, as opposed to its raw data. Thus, it is a measure of “amount of information” contained in an image [15]. Entropy is also a statistical measure of randomness and uncertainty and can be used to characterize the texture of the images where more detailed images give higher entropy. Shannon entropy measured in bits is defined as:

(2) $$H\left(X\right)=-\sum_{i=i}^{N}P\left(x_{i}\right)\log_{2}\left(P\left(x_{i}\right)\right)$$

where *X* is a discrete random variable, $P(x_{i})$ are probability of the possible outcomes $x_{i}$, and $\log_{2}$ gives the unit of bits.

For image contrast analysis, we extracted the red channel from the RGB images, as red is the dominant color for the small intestinal tissue. The co-occurrence matrix [16] is needed to calculate the contrast, which is computed as a histogram of co-occurring red channel values at a given offset, representing how often pairs of pixels with a specific value and offset occur in an image. One pixel was used as pixel pair distance offset and zero radian was used for the pixel pair angles. By using the co-occurrence matrix, the contrast is computed by:

(3) $$\mathrm{contrast}=\sum_{i}\sum_{j}G_{i,j}{\left(i-j\right)}^{2}$$

where $G_{i,j}$ is the ith row and jth column element in the co-occurrence matrix *G*.

### 2.6. CNN Architecture

CNN was chosen due to its excellent performance in image analysis, and the ability to automatically extract features, allowing for accurate differentiation between images. Through the convolutional layers, convolution is applied on the input image with kernels, making it possible to capture the spatial features of the images, resulting in feature maps. The pooling layers reduce the size of the data output so that it is easier to process further [17]. The last dense layer performs the final classification in the CNN model; in our case, there are 18 classes, “0” to “17” corresponding to “0” to “I-4, R-4”, as shown in Figure 1.

**Figure 2:**
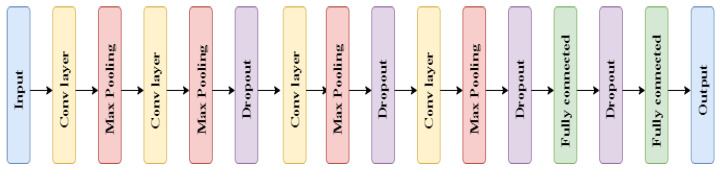
CNN architecture.

Deciding which CNN architecture performs best can be difficult, as the performance depends on the input data and the problem we seek to solve. Hence, to find the best fit for our task, we explored a few possible structures; traditional sequential CNN, decision-level fusion CNN, Bayesian CNN and ResNet50 designed by He et al. [7]. All the CNN architectures were built using Keras [18] TensorFlow as a back-end, all training were performed for 100 epochs, and Adam was used to minimize the cross-entropy loss. The traditional CNN architecture used is as follows:

Parameters used in the CNN architecture, described in Figure 2, are shown below:

- A kernel size of 3, padding “same” and l2 kernel regularizer were used for each of the convolutional layers. Hyperparemeter l2 values tested: $10^{-3},10^{-4},10^{-5}$;
- Max pooling layer of size 2 was used;
- Hyperparameter dropout values tested: 0.1, 0.2, 0.3;
- Activation function “RELU” was used for both the convolutional and the fully connected layers;
- The “Softmax” activation function was used for the last classification layer, with 18 units corresponding to the 18 classes.

The number of units for the convolutional layers were multiplied by 1, 2, 3, 4 for the 4 convolutional layers, where the initial number of units tested was: 16, 32, 64, 128, respectively. During the training, batch sizes of 32, 64, 128 were evaluated and learning rate values of 0.001 and 0.0001 were tested.

**Figure 3:**
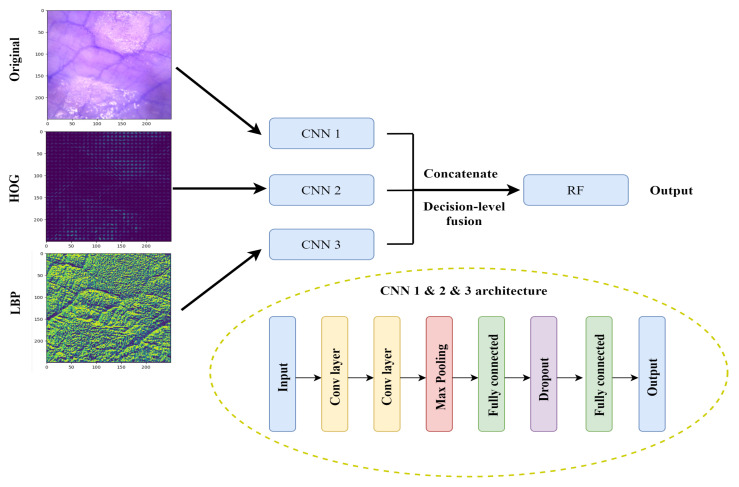
Block diagrams of decision-level fusion and CNN architecture involved.

In addition to the traditional CNN architecture in Figure 2, we created and tested a decision-level fusion framework for this multi-class classification task (Figure 3). Instead of having one input dataset, we created two additional datasets, namely histogram of oriented gradients (HOG) and local binary patterns (LBP).

HOG has been widely used in computer vision and object detection. It counts occurrences of gradient direction in a local portion of an image. HOG computes the gradient values, the gradients mainly appears at the edge of different tissue structures. The gradient direction may contain information about the blood vessels and surface structures on the small intestine. The decision-level fusion model supplemented the features extracted from the original RGB images and the HOG features to obtain a more accurate extraction of edge information. The extraction of HOG features are described in Algorithm 1.
**Algorithm 1:** HOG feature extraction.**Input:** Image dataset with RGB colors.**Step 1:** Compute the gradient of each pixel of the image, number of orientation bins used was 9.**Step 2:** Divide the images into cells, 8 × 8 pixels form a cell, and compute gradient histograms of each cell.**Step 3:** 2 × 2 cells form a block, and normalize gradient histograms across blocks.**Step 4:** The feature descriptors of all blocks are then flattened into a feature vector.**Output:** HOG features.

LBP is a powerful feature for texture classification. Earlier studies determined that combining LBP with HOG improves the detection performance considerably on some datasets [19]. The ability in extracting the texture feature enhances the information going into the model. Algorithm 2 describes the LBP feature extraction process.
**Algorithm 2:** LBP feature extraction.**Input:** Image dataset with RGB colors.**Step 1:** Convert the RGB images to grayscale images.**Step 2:** Set parameter number of circularly symmetric neighbour points to 8.**Step 3:** Set parameter radius of circle to 1.**Step 4:** Calculate the LBP feature.**Output:** LBP features.

Both HOG and LBP feature extraction were performed using the Scikit-image (skimage) library [20] in Python. The final decision-level fusion model is composed of three CNN architectures with the original RGB images, HOG images, and LBP images, as respective inputs. All three image representations were trained individually in separate CNN architectures, resulting in a combination of color, edge, texture, and local structure feature collection. Moreover, they were classified individually based on the different features extracted and the classification probability distributions were then concatenated together. The parameters used and hyperparameters tested for the architecture described in Figure 3 were the same as for the traditional CNN architecture described earlier. Furthermore, conventional classifier random forest (RF) [21] were chosen to perform the final classification. The number of trees in the forest tested was 100 and 300.

The pretrained model ResNet50 was imported from Keras application library [18], and all layers except the last block were used directly without any modifications. The last block was kept trainable in order to fine-tune the model to learn from our dataset. A global average pooling layer was added to the end of the ResNet50 model to connect the dimensions of the previous layers with the new layers. One dense layer was added after the pooling layer with dropout 0.2 before the final classification.

Data augmentation was applied for all models to increase the diversity of the dataset without having to collect more images, helping to improve the model performance and prevent the model from over-fitting. The “ImageDataGenerator” from the Scikit-learn library [22] was used to perform real-time image data augmentation. For each of the input training images, rotation (20 degrees), zoom (ranging from 90% to 110%), horizontal and vertical flip were randomly applied.

The number of images in each of the classes are unequal and the control class contains more images than the other classes. To adjust for possible bias from the unbalanced dataset, class weights were calculated and applied to all models to account for the skewed distribution of the classes. Weights were computed by $n_{\mathrm{samples}}/\left(n_{\mathrm{classes}}*n_{\mathrm{samples in a certain class}}\right)$. By including balanced class weight, the weights were increased for the minority classes and decreased for the majority classes.

### 2.7. Bayesian CNN Architecture

The library Tensorflow-probability [23] was used to implement the Bayesian CNN architecture. Compared to the standard CNN architecture described above, the following modifications were made to take both aleatoric uncertainty (statistical uncertainty) from the data and epistemic uncertainty (systematic uncertainty) of the model into account.

Instead of normal 2D convolution and dense layers, 2D convolution and dense layers with reparameterization were used, which creates an output that is drawn from a Gaussian distribution. The function that calculates the outputs uses a reparameterization estimator, which performs a Monte Carlo approximation of the distribution integrating over the kernel and bias [24]. For each replacement of the Conv2D layer with the 2D convolution reparameterization layer, there is a doubling of the number of parameters. The doubling is due to the replacement of the weight of parameters from a single value into a value that is drawn from a normal distribution with two parameters, namely, the mean and the standard deviation.

Negative log-likelihood was used as the loss function instead of categorical cross entropy, to maximize the likelihood estimate of the mean and standard deviation of the weights. We tried to maximize the probability of choosing a correct class by minimizing the negative log-likelihood, where we find lower loss with better model predictions.

To evaluate the model, several predictions were made on each of the test images instead of only once, as we did with the standard CNN architecture. A total of 300 predictions for each image were chosen for this model. From the predictions, a prediction probability distribution was created for each of the test images. One important aspect of Bayesian CNN is the output uncertainty of the model prediction. We studied the mean and the 95% confidence interval for the certainty of correct classification of the 18 classes.

### 2.8. Model Evaluation Metrics

Accuracy, precision, and recall were used to determine the performance of the model. Accuracy is the ratio of correctly predicted images to the total test images. For all models, the total image data were split into training, validation and test dataset by 60%, 20%, and 20%. Function “train_test_split” from Scikit-learn library [22] was used to randomly split the dataset. The total dataset was shuffled before splitting. Macro-averages were used in calculation of the precision and recall scores, where the metrics were calculated independently for each class before averages were calculated. The overall accuracy, precision and recall were determined as follows:

$$\mathrm{Accuracy}=\frac{\mathrm{TP}+\mathrm{TN}}{\mathrm{TP}+\mathrm{FP}+\mathrm{FN}+\mathrm{TN}}$$

where the model performance depends on true positives (TP), true negatives (TN), false positives (FP), and false negatives (TN).

$$\mathrm{Precision}=\frac{\mathrm{TP}}{\mathrm{TP}+\mathrm{FP}}\;\mathrm{Recall}=\frac{\mathrm{TP}}{\mathrm{TP}+\mathrm{FN}}$$

where precision is the probability of a positive test given that the image belongs to a specific class (images that were correctly classified as positive out of all positives) and recall is the probability of a negative test given that the image does not belong to a specific class.

### 2.9. Explainable AI—Shapley Additive Explanations (SHAP) Method

SHAP assigns each pixel of the image an importance value (Shapley value) for a particular decision, explaining how to change from the base value that would be predicted if no features were known, to the current output [9]. A pixel having a positive Shapley value indicates that the specific pixel contributes positively to the correct classification. From the distribution of Shapley values, we obtain an understanding of how different areas of the small intestine contributes to the classification.

## 3. Results

**Figure 4:**
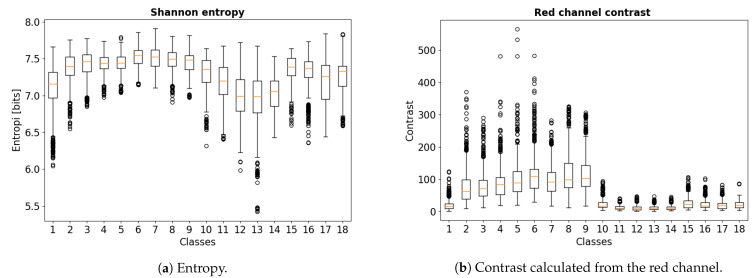
Boxplot of calculated Shannon entropy and red channel contrast values.

A total of 10 pigs were used in this study. As expected, there are considerably overlap between the different classes on both entropy and contrast. As shown in Figure 4a, the overall entropy is higher for all ischemia cases compared to the control and the reperfusion cases, where they appear to have a greater degree of disorder and thus appear to contain more “information”. The entropy is then reversed by the onset of reperfusion, where all reperfusion cases after three hours of ischemia seems to have a better reversal than reperfusion after four hours. This observation associates with findings from an earlier study suggesting that three hours of full ischemia followed by reperfusion is the upper limit for viability in the porcine intestinal ischemia model [25]. Moreover, there appears to be a clear separation between classes 10–14 (three hours ischemia followed by five hours reperfusion) and classes 15–18 (four hours ischemia followed by four hours reperfusion).

As shown in Figure 4b, the contrast of the red channel had higher values in all the ischemia cases. The intestinal segments subjected to three hours of ischemia followed by five hours of reperfusion showed an overall lower contrast. The difference between classes 10–14 and classes 15–18 is also clear in the analysis of contrast.

**Figure 5:**
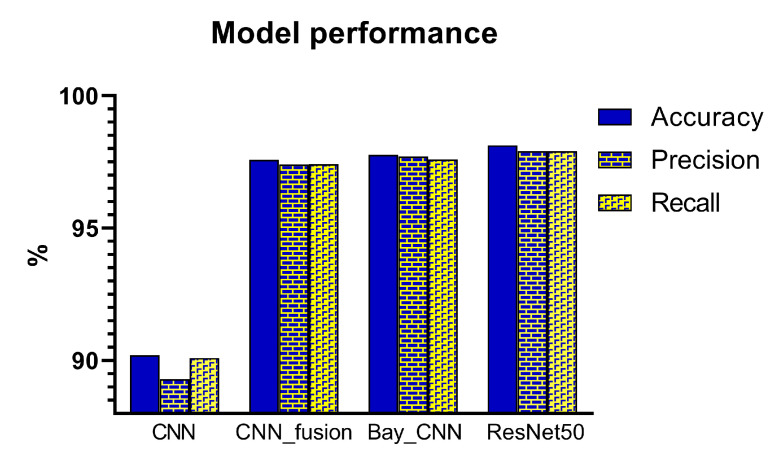
Model performance of the test dataset for 18 classes classification of different ischemia-reperfusion injury degrees of the small intestine.

As a baseline classification, a traditional CNN architecture was implemented. The architecture is shown in Figure 2, where the original RGB images alone were used as the model input. To evaluate whether a decision-level fusion approach could achieve better performance than the traditional CNN model, original RGB images combined with HOG and LBP images as the model input were evaluated. Single representations of the three image datasets were trained using separate CNN architectures, and the results of these three CNN classifiers were then concatenated and fed into a RF classifier, as shown in Figure 3. Figure 5 compares the performance from the test dataset of different models. ResNet50 outperformed all other models with an average accuracy of 98.12%, followed by the Bayesian CNN and CNN with decision-level fusion with an average accuracy of 97.76% and 97.57%, respectively. The traditional CNN model only achieved an accuracy of 90.20%.

**Figure 6:**
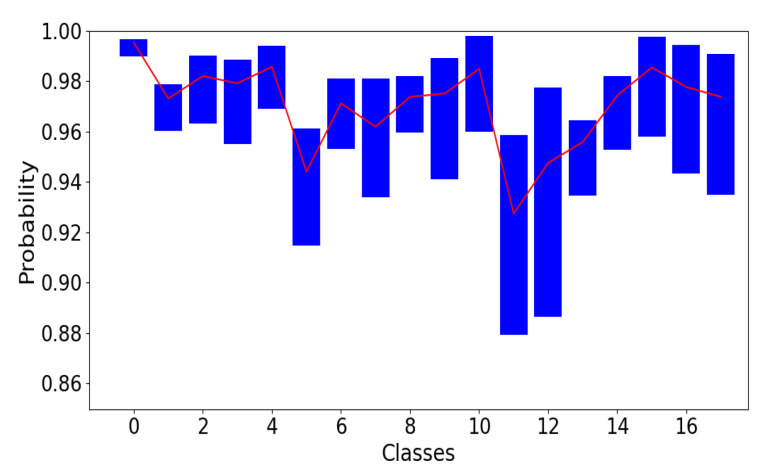
The 95% confidence interval for correctly predicted images together with the mean. The upper limit is the average value of 97.5th percentile, and the lower limit is the average value of the 2.5th percentile averaged over 300 predictions and all test images in the same class. Confidence interval for prediction probability for the 18 classes. Classes “0” to “17” corresponds to “0” to “I-4, R-4” in Figure 1.

The implementation of Bayesian CNN gives us the possibility to quantify the uncertainty of the model. Consider one input image, which has been classified to class “10” with a probability of 99%, but the uncertainty could also be as high as 10%, therefore, we cannot fully trust the model performance without uncertainty measurements. To evaluate the model uncertainty, we show the mean and the average 95% confidence interval for model prediction certainty in Figure 6.

The model predicts each of the test images 300 times instead of just once. Of the 300 predictions, in one run the model may predict an image as class 11 with almost 96% probability, in another run, it may predict the same image with only 89% probability. Those classes with wide 95% confidence intervals and lower values are those the model struggles with predicting correctly with high certainty.

**Figure 7:**
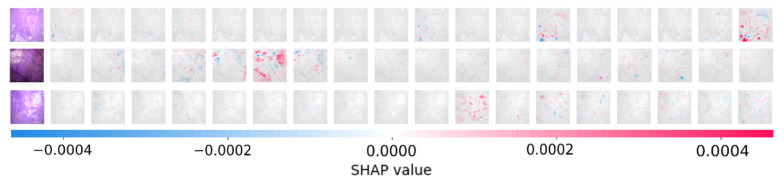
Visualization of positive and negative contributions to the classification by the SHAP method. The three images belong to classes 18, 6, and 11, respectively.

Figure 7 shows the output from the SHAP method, where it attempts to explain the model decisions by highlighting different pixels on the image. Red pixels represent positive Shapley values, which contribute to increase the possibility of being classified to the correct corresponding class. Blue pixels represent negative Shapley values that reduce the probability of being classified to the correct class. The colored images on the left side of Figure 7 are the original images. The correct classes for the three example images are: class 18, 6, and 11. The grey images in each row represent the 18 classes. All three images were correctly classified, as we can see from the first row, the 18th grey image (corresponding to class 18—4 h ischemia followed by 4 h reperfusion) had the largest positive contribution, the red color marked areas correspond to the same areas on the original image. From Figure 7, we are not only seeing which areas on the small intestine images contributed positively and negatively, we can also see which classes are similar to the target correct class, where some areas also contribute positively but not as much. Images with similar injuries look similar, SHAP outputs show where on the image does the model utilize as important features, providing a method in verifying that our model did not consider undesirable noise features as important basis for classification.

## 4. Discussions

By combining microscopic images from intestinal segments in a pig model of mesenteric ischemia-reperfusion with machine learning models, we were able to detect whether the segments were ischemic or reperfused, and could also assess how long the segments had been ischemic or reperfused, with a high accuracy.

This addresses the well known challenge of viability assessment and selection of resection margins following ischemia-reperfusion in the intestines. The standard clinical methods to decide which parts should be resected can require waiting for up to 48 h for a second look surgery, and uncertainty can be high. When fully perfused or fully necrotic, the intestine can be easy to identify by visual inspection, reperfused or partially reperfused intestine with return of color and movement can be harder to assess with respect to viability [25]. Accordingly, there is a high medical need for methods that can accurately predict intestinal viability after ischemia-reperfusion injury.

The method of using surface images together with machine learning is fast, easy-to-use, and can potentially be used as a tool to assist the surgeon with decision-making during surgery. As the intestinal wall is thin and some of the intestinal layers are semi-transparent, visual light contains information not only about the serosal surface, but of the subserosa and muscularis, as well as distribution and size of blood vessels in these layers. We chose to investigate both ischemia and reperfusion, as intestinal injury is caused not only by ischemia but that injury is aggravated following reperfusion [26,27,28].

Transfer learning using ResNet50 achieved an accuracy of 98.12%. This model architecture has the largest number of layers, and it was pre-trained on a dataset containing one million images [7]. ResNet50 showed a very good ability in both capturing the features and generalizing itself on our dataset. In addition, the skip connections allow alternate shortcut paths for gradient to flow through, which greatly reduce the risk for the vanishing gradient problem [29]. For clinical questions involving medical images, transfer learning is worth exploring, since it is both time saving and has less demand for training on a large dataset. This can be of a great advantage when working with medical images, as collecting data from patients can be challenging and time-consuming.

Deep learning models lack transparency and can sometimes be difficult to trust. Probability theory can be adopted to express uncertainty and noise associated with the model, and inverse probability allows us to infer unknown quantities. Bayes rule informs us how to perform inference about hypotheses from our dataset and help us to analyze the model prediction uncertainty [30]. The key difference between a standard CNN and a Bayesian CNN is that the weight and biases in the network has a probability distribution attached to them instead of a single deterministic value representing the weight and bias. For each of the images classified, we run through the prediction several times, which gives us multiple output values. From the output values, we can find the uncertainty and confidence intervals. With low-quality, noisy images or images that are completely different from the rest, the output will have a more “uniform-like” distribution, which could be interpreted as high uncertainty as the model classifies the same image to a different class each time. High-quality images combined with a well-trained model would give us the same prediction of the true class, as the model tends to draw the same conclusion each time on the same image. This could be interpreted as the model being able to extract the real features which contributes positively to the correct classification. Ultimately, the Bayesian CNN achieved the second best performance while providing uncertainty measurements. These reveals how confident the model is when assigning a probability value to a certain class.

As shown in Figure 6, the overall prediction certainty agrees with what we see from the images, for example, those classes which represent ischemia followed by reperfusion are more difficult to distinguish from one another. As we can see from Figure 6, class 11 and 12 corresponding to 3 h ischemia followed by 3 and 4 h reperfusion, had the lowest mean certainty and widest confidence interval.

CNN with decision-level fusion using three different image representations fell to the third place in performance. Merging different image representations and training them in separate CNN models allow for more specific and accurate extraction of features, leading to a better result compared to the conventional CNN model.

It can be challenging to understand how the machine learning models arrive at the output classification decision. Machine learning algorithms are becoming more and more complex, with increasing numbers of layers in a single model [31]. The result can be black-box models where the interpretations of the relation between different variables can be challenging to explain [32]. This is known as the interpretability problem [33]. In order to address this problem, we explored several explainable AI methods, including SHAP [9], gradient-weighted class activation mapping (GradCam), local interpretable model-agnostic explanations (LIME), and explain it like I am 5 (Eli5). The SHAP method was found to be the most suitable and best-performing method for our dataset. Therefore, the SHAP method was applied in order to explain how the decisions were made by the model.

Accompanying the CNN performance, one of the most important findings was that the two image parameters entropy and contrast of the red channel clearly indicated a systematic image change in the ischemia and reperfusion phase. As these two parameters are quite independent, this indicates that there is important information at both structure level and color level in the images that can be used by the CNN models for classification. The link between the two image parameters and the highlighted areas on the SHAP output are of interest, where we might be able to connect statistical image parameters with deep learning. For instance, including the statistical image parameters as features in a hybrid machine learning model may improve the model performance.

Both the SHAP method and the statistical image parameter analysis method provide information about the intestinal images. From Figure 7, we can see that some of the blood vessels were highlighted, which are some of the patterns that CNN recognizes naturally. The larger blood vessels near the small intestine surface contribute both to higher values of entropy and contrast. In the future, we plan to study the characteristics behind the highlighted areas on a SHAP output plot, where possibly important features and changes during ischemia-reperfusion injury on the small intestine can be revealed, which are not yet known.

There are some limitations to this study. First, the small number of pig subjects that we used, which may lead to a limited generalization of the model, where both within group variance and between group variance may not be fully covered. We observed variations both between pigs and between different locations in the same small intestine segment. Another limitation is the segmental warm full ischemia and reperfusion model that we used, which does not cover all clinical conditions. Different clinical conditions, such as partial occlusion, may lead to different physiological behaviors, causing variations in appearance of the small intestine. With respect to sources of measurement error, due to the semi-transparent nature of the small intestine, light from surrounding light sources may have entered the site causing variations in amount of light in the images. When downsizing the images, important information might have been lost, which is a limitation that was chosen based on the available computer memory size. Moreover, images from all individual pigs and intestinal segments were mixed first, before separating to training, validation, and test datasets. Evaluation of model performance and generalizability might be weakened as images from the same intestinal segment may be included in different batches during both training and testing, which might lead to over-fitting. The results might be biased as images from the same pig can fall into both the training and the test dataset. Our method needs to be validated on a larger dataset and preferably with human intestine, to assess the realistic potential in this method.

The overall results suggest that a combination of microscopic images taken from the small intestine surface can potentially be used as an objective, fast, and accurate method to assess ischemia-reperfusion injury on the small intestine. This proposed technique does not require clinical and technical experience, and due to the non-invasive nature of the method, it can be relatively easy to attain clinical approval with respect to sterile requirements and electrical safety.

## 5. Conclusions

In this study, we investigated whether microscopic images taken from the surface of the small intestine in vivo in a pig model, combined with deep learning, can be used to assess ischemia-reperfusion injuries. Different deep learning models were evaluated and compared, ResNet50 outperformed the Bayesian CNN, CNN with decision-level fusion and traditional CNN. The uncertainty estimation provided by the Bayesian CNN model can be important in clinical settings. In combination with the explainable AI-SHAP method, not only can we assess the viability state of the small intestine, at the same time, we can explain what regions of the intestine the model used as a basis for decision making. A clear indication of systematic changes on the images in the ischemia and reperfusion phases was found in the entropy and contrast properties of the images. This work lays the cornerstone for a planned future study where we aim to make use of a much larger dataset with images of the intestine surface captured either manually during open surgery or from laparoscopic video taken from patients who undergo laparoscopy surgery to evaluate our method.

## Data Availability

Data are available from the authors upon reasonable request.
